# Design of Composite Photocatalyst of TiO_2_ and Y-Zeolite for Degradation of 2-Propanol in the Gas Phase under UV and Visible Light Irradiation

**DOI:** 10.3390/molecules191016477

**Published:** 2014-10-13

**Authors:** Takashi Kamegawa, Yasushi Ishiguro, Ryota Kido, Hiromi Yamashita

**Affiliations:** 1 Division of Materials and Manufacturing Science, Graduate School of Engineering, Osaka University, 2-1 Yamadaoka, Suita, Osaka 565-0871, Japan; 2 Nanoscience and Nanotechnology Research Center, Osaka Prefecture University, 1-2 Gakuencho, Nakaku, Sakai, Osaka 599-8570, Japan; 3 Elements Strategy Initiative for Catalysts and Batteries (ESICB), Kyoto University, Katsura, Kyoto 615-8520, Japan

**Keywords:** photocatalyst, TiO_2_, Y-zeolite, composite, hydrophobic modification, visible light, decomposition of 2-propanol gas

## Abstract

Hydrophobic Y-zeolite (SiO_2_/Al_2_O_3_ = 810) and TiO_2_ composite photocatalysts were designed by using two different types of TiO_2_ precursors, *i.e.*, titanium ammonium oxalate and ammonium hexafluorotitanate. The porous structure, surface property and state of TiO_2_ were investigated by various characterization techniques. By using an ammonium hexafluorotitanate as a precursor, hydrophobic modification of the Y-zeolite surface and realizing visible light sensitivity was successfully achieved at the same time after calcination at 773 K in the air. The prepared sample still maintained the porous structure of Y-zeolite and a large surface area. Highly crystalline anatase TiO_2_ was also formed on the Y-zeolite surface by the role of fluorine in the precursor. The usages of ammonium hexafluorotitanate were effective for the improvement of the photocatalytic performance of the composite in the degradation of 2-propanol in the gas phase under UV and visible light (λ > 420 nm) irradiation.

## 1. Introduction

Titanium dioxide (TiO_2_)-based photocatalytic materials have been used for the decomposition of undesired and harmful organic compounds in the air and water [1,2,3,4,5,6,7]. Electron-hole pairs formed under light irradiation by using a suitable light source play significant roles in the degradation of organic compounds into CO_2_ and H_2_O. TiO_2_-based photocatalytic materials are also continuously researched for their importance in relation to the utilization of light energy for the synthesis of chemicals, the production of clean energy, *etc.*, under carefully-controlled conditions [8,9,10,11,12,13]. Coating technologies for TiO_2_-based photocatalytic materials open the way for the utilization of unique functions, such as photocatalytic activity, the self-cleaning effect and photoinduced superhydrophilicity [3,14,15,16,17]. By increasing the awareness of environmental issues, purification of diluted organic contaminants in water and air is becoming an increasingly important agenda. TiO_2_-based photocatalytic materials have potential to solve the problem of air pollution by volatile organic compounds in our living spaces, *i.e.*, the cause of sick house syndrome emitted from interiors. However, photocatalytic performance of TiO_2_ is insufficiently utilized in our living spaces, due to the limitation of the amount of light in the UV region. Bare TiO_2_ can only absorb UV light, which also corresponds to *ca*. 3% of the energy of natural solar light. Therefore, many efforts have been devoted to the design of highly efficient photocatalysts, which can work under not only UV, but also visible, light irradiation. Doping of various heteroatoms, e.g., carbon, nitrogen, sulfur and transition metals (Cr, V, Fe, *etc.*), into TiO_2_ was previously reported and enabled the use of light in the visible region [18,19,20,21,22,23,24,25]. The modification of the TiO_2_ surface by metals, metal ions and chlorides is also another method for realizing the sensitivity to visible light [26,27,28]. Utilization of visible light is also achieved by the anchoring of phenolic compounds on the TiO_2_ surface by the formation of surface complexes [12,13].

On the other hand, adsorbents, such as activated carbon, are often used as a disposable material for the removal of diluted organic contaminants in water and air. The combination of adsorbent and TiO_2_ photocatalyst is intensively studied for the design of efficient photocatalytic systems with specific functions [6,29,30,31,32,33,34,35,36]. Adsorption and enrichment of organic contaminants around combined TiO_2_ from air and water play crucial roles in the photocatalytic decomposition process and lead to the efficient removal of diluted organic contaminants in the air and water. The combination of TiO_2_ and silicate materials, such as zeolite, mesoporous silica and clay minerals, is achieved by applying different methods, such as wet impregnation and sol-gel processes [29,30,31,32,33,34,35,36]. The physical and chemical properties of the composites, especially the surface properties, such as hydrophilicity/hydrophobicity or surface charge, strongly affect the photocatalytic performance. In our former works, surface modification from a hydrophilic to more hydrophobic state was successfully achieved by the grafting of the fluorine group containing silylation reagents on zeolite and the mesoporous silica surface [36]. The effect of the direct and selective modification of the TiO_2_ surface on mesoporous silica by graphene on the photocatalytic performance in water purification was also shown in a previously reported paper [37]. In the case of composite systems, photocatalytic performance in water and air purification relies on the surface properties. In the present work, we designed a composite system of hydrophobic Y-zeolite (SiO_2_/Al_2_O_3_ = 810) and TiO_2_ by using two different types of TiO_2_ precursors (titanium ammonium oxalate ((NH_4_)_2_[TiO(C_2_O_4_)_2_]) and ammonium hexafluorotitanate ((NH_4_)_2_[TiF_6_]). We mainly focus on the application of the composite in the decomposition of 2-propanol in the gas phase as a model contaminant of air under UV and visible light (λ > 420 nm) irradiation.

## 2. Results and Discussion

### 2.1. Characterization of TiO_2_/Y-Zeolite Composite Photocatalysts

**Figure 1:**
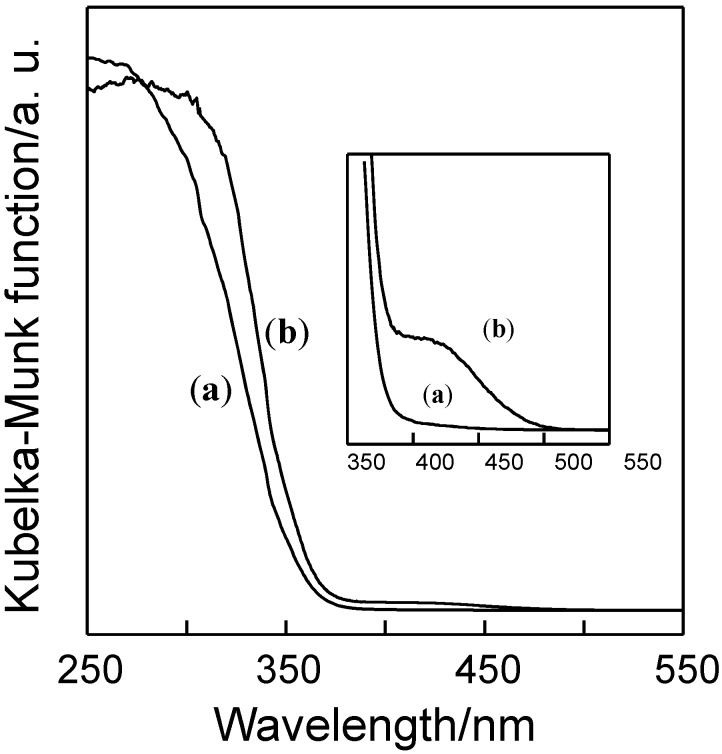
UV-Vis absorption spectra of (**a**) AO-TiO_2_/Y and (**b**) AF-TiO_2_/Y.

Figure 1 shows the UV-Vis absorption spectra of AO-TiO_2_/Y and AF-TiO_2_/Y, which were prepared by a combination of Y-zeolite and (NH_4_)_2_[TiO(C_2_O_4_)_2_] or (NH_4_)_2_[TiF_6_] as a TiO_2_ source, respectively [35]. Both samples exhibited the typical absorption in the UV light region corresponding to the band gap energy of TiO_2_ particles loaded on Y-zeolite. The absorption band edge of TiO_2_ was obviously changed by the quantum-size effect [38]. The blue shifts of absorption spectra suggest that TiO_2_ nanoparticles are successfully loaded on the Y-zeolite surface with a dispersed state. The small differences in the absorption spectra of AO-TiO_2_/Y and AF-TiO_2_/Y indicate the differences of the TiO_2_ particle size and crystallinity formed on the Y-zeolite surface [39,40]. The powder color of AO-TiO_2_/Y was a simple white, while AF-TiO_2_/Y was a clear yellow powder. As shown in Figure 1 (inset), AF-TiO_2_/Y exhibited visible light absorption from 400 to 500 nm. This visible light absorption was estimated to be induced by the doping of nitrogen and fluorine into TiO_2_ during the decomposition of (NH_4_)_2_[TiF_6_] in the calcination process. No visible light absorption was attained by use of (NH_4_)_2_[TiO(C_2_O_4_)_2_], although both precursors contain a nitrogen source (ammonium cation). The existence of fluorine might induce the encapsulation of nitrogen within TiO_2_ formed on the Y-zeolite surface by keeping a charge balance of each component (Ti^4+^, O^2−^, N^3−^ and F^−^) during the calcination in the air [41,42].

**Figure 2:**
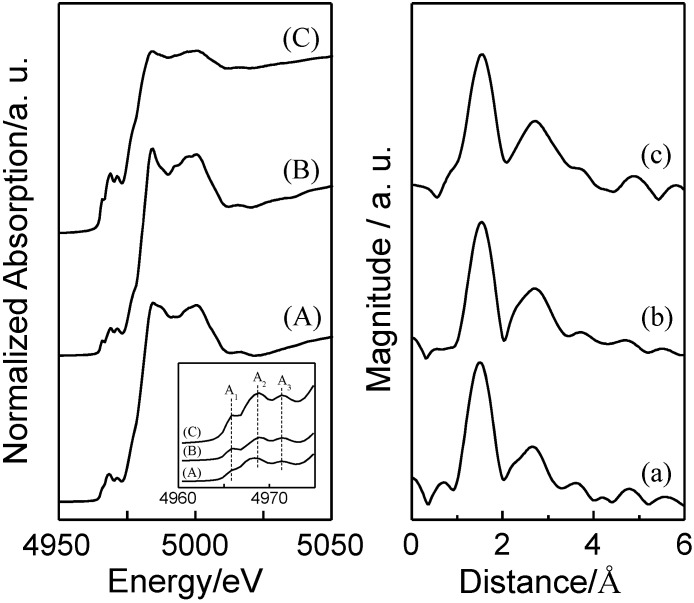
(A–C) XANES and (a–c) Fourier transforms of EXAFS spectra of (A,a) AO-TiO_2_/Y, (B,b) AF-TiO_2_/Y and (C,c) TiO_2_ (anatase).

The state of TiO_2_ in each sample was investigated by Ti K-edge X-ray absorption fine structure (XAFS) measurements. Figure 2A–C shows the X-ray absorption near edge structure (XANES) of AO-TiO_2_/Y, AF-TiO_2_/Y and the reference sample (anatase TiO_2_). The XANES spectrum of anatase TiO_2_ exhibited several pre-edge peaks (A_1_, A_2_, and A_3_) from 4960 to 4975 eV. The A_1_ peak is assigned to an exciton band or the 1s to 1t_1g_ transition. The A_2_ and A_3_ peak is attributed to the 1s to 3d transition, as well as being also assigned to the 1s to 2t_2g_ and 1s to 3d transitions, respectively [25,43,44]. AO-TiO_2_/Y and AF-TiO_2_/Y exhibited well-defined three pre-edge peaks, which were similar to those for anatase TiO_2_ as a reference. These results indicated that anatase TiO_2_ was formed on the Y-zeolite surface without relying on the differences of TiO_2_ precursors. In the Fourier transforms of extended X-ray absorption fine structure (EXAFS ) spectra (Figure 2a–c), peaks due to the existence of oxygen neighbors (Ti-O) and the Ti neighbors (Ti-O-Ti) were observed at *ca*. 1.8 and between 2.0 and 3.0 Å (without phase-shift correction), respectively [39,40]. The clear peak due to the existence of the Ti-O-Ti bond indicated the formation of aggregated large TiO_2_ particles with octahedral coordination. AF-TiO_2_/Y exhibited a relatively intense peak compared to that of AO-TiO_2_/Y, showing the formation of TiO_2_ with relatively high crystallinity by using (NH_4_)_2_[TiF_6_] as a precursor.

**Figure 3:**
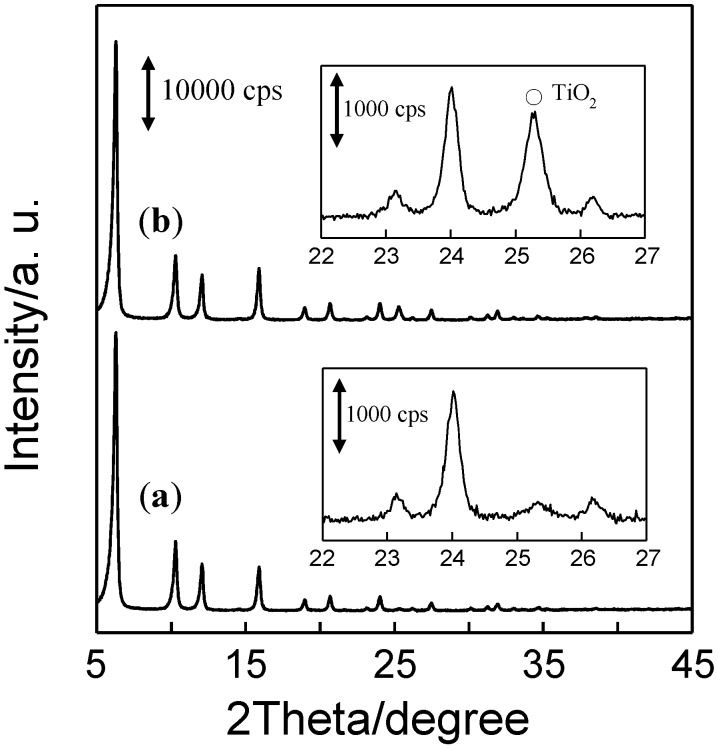
XRD patterns of (**a**) AO-TiO_2_/Y and (**b**) AF-TiO_2_/Y.

The XRD patterns of AO-TiO_2_/Y and AF-TiO_2_/Y are shown in Figure 3. The diffraction peaks attributed to the framework structure of Y-zeolite (5° < 2θ < 45°) were clearly observed in both samples. The typical peak assigned to the (101) reflection of the TiO_2_ anatase phase is observed at around 2θ = 25° in the XRD measurement. AF-TiO_2_/Y showed an intense peak in this region, while AO-TiO_2_/Y only showed a small peak, except for the peaks due to the Y-zeolite. The crystallinity of TiO_2_ formed on the Y-zeolite surface was affected to a large degree by the differences of the TiO_2_ precursors. It has been reported that crystallization of TiO_2_ was enhanced by the role of the fluorine ion. The addition of the fluorine ion in the hydrolysis process of titanium isopropoxide realized the good crystallinity of TiO_2_ [40,41]. AF-TiO_2_/Y have thus relatively high crystallinity by the effect of the contained fluorine in (NH_4_)_2_[TiF_6_] used as a precursor.

**Figure 4:**
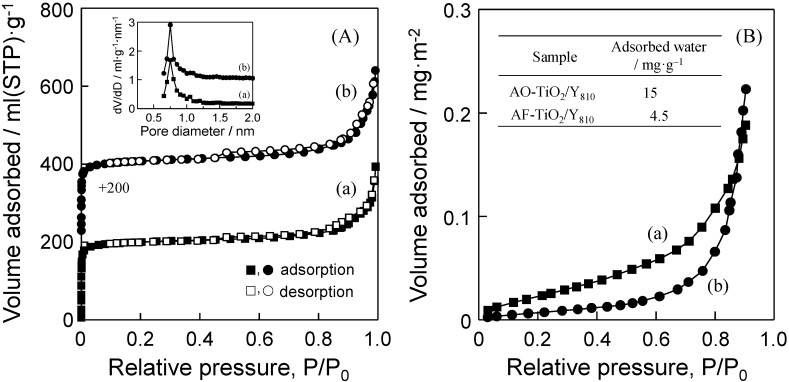
(**A**) Nitrogen adsorption-desorption isotherms at 298 K and (**B**) water adsorption isotherms at 298 K of (a) AO-TiO_2_/Y and (b) AF-TiO_2_/Y. The insets of (A) and (B) show the pore size distribution curves and the adsorbed amount of water at around P/P_0_ = 0.2, respectively.

The textural properties of AO-TiO_2_/Y and AF-TiO_2_/Y were investigated by the measurement of nitrogen adsorption-desorption isotherms at 77 K. As shown in Figure 4A, AO-TiO_2_/Y and AF-TiO_2_/Y exhibited typical type I isotherms with a steep increase in the adsorbed amount of nitrogen at the low relative pressure region (P/P_0_ < 0.01). The BET surface area of samples was determined to be 664 m^2^/g (AO-TiO_2_/Y) and 645 m^2^/g (AF-TiO_2_/Y), respectively. The pore size distribution curve also showed a peak at around 0.75 nm. These kinds of TiO_2_ precursors hardly affect the structure of Y-zeolite.

The investigations of the surface hydrophilic and hydrophobic nature of both samples were also carried out by measurement of water adsorption isotherms at 298 K. Figure 4B shows the water adsorption isotherms of AO-TiO_2_/Y and AF-TiO_2_/Y. The adsorbed amount of water on AF-TiO_2_/Y was quite small up to relative pressure, P/P_0_ = 0.8. The inset of Figure 4B shows the adsorbed amount of water at around P/P_0_ = 0.2 on both samples. The adsorbed amount of water on AF-TiO_2_/Y was less than a third of that on AO-TiO_2_/Y, showing the good surface hydrophobic property of AF-TiO_2_/Y.

**Figure 5:**
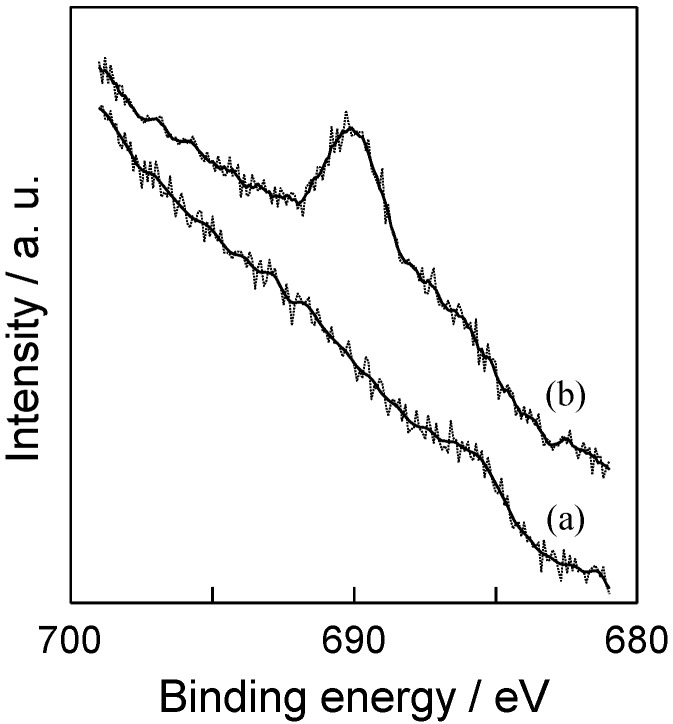
F_1s_ XPS spectra of (a) AO-TiO_2_/Y and (b) AF-TiO_2_/Y.

In the case of zeolite, the surface hydrophilic and hydrophobic nature depends strongly on the SiO_2_/Al_2_O_3_ ratio of samples. As the SiO_2_/Al_2_O_3_ ratio of zeolite increases, the adsorbed amount of water becomes small. Y-zeolite with high SiO_2_/Al_2_O_3_ ratio (SiO_2_/Al_2_O_3_ = 810), which has a good hydrophobic nature in the series of commercially available Y-zeolite, was adopted for the preparation of AO-TiO_2_/Y and AF-TiO_2_/Y. The surface property of samples significantly changed depending on the kinds of TiO_2_ precursors. By using (NH_4_)_2_[TiF_6_] as a precursor, the improvement of the surface hydrophobic property of the TiO_2_-zeolite composite was achieved in a brief preparation process. Accompanying the generation of hydrofluoric acid and ammonia gas, the decomposition of (NH_4_)_2_[TiF_6_] to TiO_2_ gradually occurs above 473 K [45]. Generated hydrofluoric acid gas might be reacting with the surface hydroxyl groups of Y-zeolite and the formation of fluorinated groups, which make the surface of AF-TiO_2_/Y quite hydrophobic. The presence of fluorine moieties (Si-F) on the surface of AF-TiO_2_/Y were confirmed by F_1s_ XPS analysis. As shown in Figure 5, AF-TiO_2_/Y exhibited a weak peak at around 690 eV, while no peak was observed in the case of AO-TiO_2_/Y. This peak was assigned to the covalent F atoms, indicating the formation of Si-F through the reaction of surface hydroxyl groups and generated hydrofluoric acid gas [36]. The peak attributed to the presence of the F^−^ ion doped into TiO_2_ is also observed in the same region [42]. However, no peak was observed in the N_1s_ XPS analysis of AF-TiO_2_/Y. The concentration of nitrogen in AF-TiO_2_/Y was below the detection limitation. Considering these obtained results, the amount of nitrogen and fluorine doped into TiO_2_ on the Y-zeolite surface was estimated to be so small, while AF-TiO_2_/Y exhibited clear absorption in the visible light region. These results suggested that a large part of fluorine exists on the Y-zeolite surface and leads to the good surface hydrophobicity. In fact, AF-TiO_2_/Y showed small a peak in FT-IR spectrum at around 3740 cm^−1^ as compared to that of AO-TiO_2_/Y. This peak is assigned to the surface hydroxyl group of Y-zeolite.

### 2.2. Photocatalytic Performance of TiO_2_/Y-Zeolite Composite Photocatalysts

**Figure 6:**
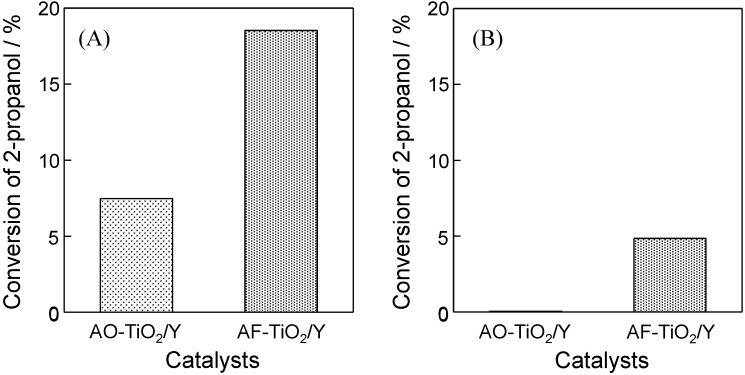
Conversion in the photocatalytic degradation of 2-propanol gas diluted in the air on AO-TiO_2_/Y and AF-TiO_2_/Y under (**A**) UV and (**B**) visible light (λ > 420 nm) irradiation (reaction time: (A) 1 h; (B) 12 h).

The photocatalytic performance of AO-TiO_2_/Y and AF-TiO_2_/Y was evaluated in the degradation of 2-propanol gas diluted in the air under UV and visible light (λ > 420 nm) irradiation. The degradation of 2-propanol in the gas phase was adopted as a model reaction. It is well known that 2-propanol was decomposed to CO_2_ and H_2_O via the formation of acetone. In the initial reaction stage, acetone was mainly formed in the gas phase. The formed acetone is fully decomposed into CO_2_ and H_2_O by the progress of the reaction time [28,36,39]. As shown in Figure 6A, AF-TiO_2_/Y exhibited a two-times higher photocatalytic activity than that on AO-TiO_2_/Y under UV light irradiation. As is obvious from the XRD measurement, AF-TiO_2_/Y contained TiO_2_ with good crystallinity, leading to an enhancement of photocatalytic performance. The crystallinity of TiO_2_ is related to various properties, such as electron conductivity, hole mobility and the electron-hole recombination probability. The good crystallinity of TiO_2_ reduces the recombination probability and enhances the photocatalytic reactions [46,47]. In the case of hydrophilic zeolite, H_2_O molecules are easily adsorbed on the surface and filled inside of pores. Organic molecules are thus not preferentially adsorbed on the surface and diffuse into the pores, resulting in lower photocatalytic activity. The noticeable improvement on AF-TiO_2_/Y was attained by the combinational effect of the hydrophobicity of the Y-zeolite support and the good crystallinity of the formed TiO_2_. In response to the light absorption property, AF-TiO_2_/Y also showed photocatalytic activity under visible light (λ > 420 nm) irradiation (Figure 6B). Utilization of (NH_4_)_2_[TiF_6_] is effective for realizing the improvement of the hydrophobicity of the support, the good crystallinity of TiO_2_, as well as the visible light sensitivity in a simple preparation process.

## 3. Experimental Section

### 3.1. Materials

Proton type Y-zeolite (SiO_2_/Al_2_O_3_ = 810) used as a support of the TiO_2_ photocatalyst was supplied by Tosoh Co. (Tokyo, Japan). Titanium ammonium oxalate ((NH_4_)_2_[TiO(C_2_O_4_)_2_]) and ammonium hexafluorotitanate ((NH_4_)_2_[TiF_6_]) were purchased from Kishida Chemicals (Osaka, Japan). 2-propanol was purchased from Nacalai Tesque Inc (Kyoto, Japan). All chemicals were used without further purification.

### 3.2. Sample Preparation

The combination of TiO_2_ and Y-zeolite (SiO_2_/Al_2_O_3_ = 810) was carried out by a conventional impregnation method [35,37]. Y-zeolite was suspended in an aqueous solution of (NH_4_)_2_[TiO(C_2_O_4_)_2_] or (NH_4_)_2_[TiF_6_] and was stirred at 323 K for 1 h. Water was then evaporated at 343 K under reduced pressure. The obtained powder was dried at 373 K for 12 h and then calcined at 773 K for 5 h in the air (heating rate: *ca*. 2.5 K/min). The content of TiO_2_ was adjusted to 10 wt % in both samples. The thus obtained samples were denoted as AO-TiO_2_/Y and AF-TiO_2_/Y, which were prepared by using an aqueous solution of (NH_4_)_2_[TiO(C_2_O_4_)_2_] and (NH_4_)_2_[TiF_6_], respectively.

### 3.3. Characterization Techniques

Nitrogen adsorption-desorption isotherms at 77 K, as well as water adsorption isotherms at 298 K were measured by a BEL-SORP max (BEL Japan, Inc., Osaka, Japan). Prior to the measurements of isotherms, each sample was degassed under vacuum at 473 K for 2 h. Diffuse reflectance UV-Vis spectra were recorded at 298 K with a Shimadzu UV-2450A double-beam digital spectrophotometer. FT-IR measurements were carried out at 298 K in transmission mode with a resolution of 4 cm^−1^ using a JASCO FT/IR-6100. Prior to FT-IR measurements, self-supporting pellets of samples were degassed at 673 K for 1 h. XPS measurements were carried out by using a Shimadzu ESCA-3200 using Mg Kα radiation. The powder XRD measurements were performed using a Rigaku Ultima IV X-ray diffractometer with Cu Kα radiation. Ti K-edge X-ray absorption fine structure (XAFS) measurements were carried out at the BL-7C facility of the Photon Factory (high energy acceleration research organization, Tsukuba, Japan). XAFS spectra were measured at 298 K in the fluorescence mode. A Si(111) double crystal was used to monochromatize the synchrotron radiation from the 2.5 GeV electron storage ring. The obtained data were examined using the analysis program (Rigaku REX2000). Fourier transformations were performed on *k*^3^-weighted EXAFS oscillations in the range 3–10 Å^−1^ to obtain the radial structure function. Thermogravimetry-differential thermal analyses for determining the decomposition temperature of (NH_4_)_2_[TiF_6_] was performed using a TG-DTA2000S (MAC Science Co. Ltd., Tokyo, Japan) from RT to 1073 K at a heating rate of 10 K/min under an air flow (50 mL/min).

### 3 4. Photocatalytic Reaction

The photocatalytic activity of samples was evaluated by monitoring the decomposition of 2-propanol gas in the air under UV and visible light irradiation. A sample (10 mg) was fixed on a glass filter in accordance with previous papers [27,28]. After pretreatment for the removal of residual organics on the surface, the gas phase in a glass reactor equipped with a flat quartz window was replaced with artificial air. 2-propanl gas (0.13 mmol) was then injected into the reactor. The irradiation of UV light was carried out by using a 200-W mercury xenon lamp (UVF-204S, San-ei Electric Co., Ltd., Osaka, Japan) under controlled light intensity (5 mW/cm^2^ at around 360 nm). Visible light (λ > 420 nm) irradiation was also performed by using the same light source through a colored filter (HOYA; L-42). The progress of the reaction was monitored by gas chromatography analysis (Shimadzu GC-14B with FID and TCD, Kyoto, Japan).

## 4. Conclusions

Comparative studies were carried out by using composite photocatalysts (AO-TiO_2_/Y and AF-TiO_2_/Y) prepared by a combination of Y-zeolite (SiO_2_/Al_2_O_3_ = 810) and two different types of precursors ((NH_4_)_2_[TiO(C_2_O_4_)_2_] and (NH_4_)_2_[TiF_6_]). AF-TiO_2_/Y, having visible light sensitivity, good crystallinity of TiO_2_ and a highly hydrophobic surface property, was successfully achieved by using (NH_4_)_2_[TiF_6_] as a precursor. Instead of the use of (NH_4_)_2_[TiO(C_2_O_4_)_2_] as a precursor, these additional and advanced functions were realized at the same time in a simple preparation process. Based on these functions, AF-TiO_2_/Y exhibited a good photocatalytic performance in the decomposition of 2-propanol in the gas phase under not only UV, but also visible, light (λ > 420 nm) irradiation.
